# Short-Term Changes in Bone Metabolism Among Transgender Men Starting Gender-Affirming Hormone Therapy: A Systematic Review and Meta-analysis

**DOI:** 10.1007/s00223-024-01296-z

**Published:** 2024-10-02

**Authors:** Daniele Tienforti, Lorenzo Marinelli, Jeroen Vervalcke, Luca Spagnolo, Federica Antolini, Andreina Bichiri, Marco Giorgio Baroni, Giovanna Motta, Guy T’Sjoen, Arcangelo Barbonetti

**Affiliations:** 1 Andrology Unit, Department of Clinical Medicine, Life, Health and Environmental Sciences, University of L’Aquila, 67100 L’Aquila, Italy; 2 Division of Endocrinology, Diabetes, and Metabolism, Department of Medical Sciences, University of Turin, Turin, Italy; 3 Department of Endocrinology, Center for Sexology and Gender, Ghent University Hospital, Ghent, Belgium

**Keywords:** Transmen, GAHT, Gender incongruence, Testosterone, BMD, Bone turnover markers

## Abstract

Transgender and gender diverse individuals experience a gender identity that differs from the sex assigned at birth. Some transgender men may request testosterone to induce virilization; however, its impact on bone health remains to be fully elucidated. The objective of this systematic review and meta-analysis was to evaluate the modifications in bone metabolism over a short-term period among transgender men initiating testosterone therapy. A systematic search was conducted in PubMed, Scopus, Web of Science, and Cochrane Library. The articles of interest had to report longitudinal evaluation conducted among transgender men, before starting testosterone and after 12 and 24 months of therapy. The analyzed parameters were BMD, calcium, phosphate, 25OHD, PTH, P1NP, BAP, osteocalcin and CTx. Mean differences with 95% coefficient intervals were combined using random effects models. Funnel plot, Egger’s test, and trim-and-fill analysis were used to assess publication bias. Fourteen studies met the inclusion criteria, including 1484 subjects. In absence of heterogeneity, BMD did not significantly change at lumbar spine, hip, femoral neck, and whole-body evaluations. Calcium, phosphate, 25OHD and PTH remained stable over time. Regarding bone turnover markers, only P1NP showed a statistically significant increase after 12 months of T therapy, in absence of heterogeneity (SMD 0.61 mcg/l; 95% CI: 0.40–0.83; *p* < 0.0001; *I*^2^ = 0%, *P*forheterogeneity = 0.48). Testosterone therapy among transgender men seems not to disrupt bone health after 12 and 24 months. A statistically significant elevation in P1NP levels after 12 months of therapy may indicate a positive anabolic effect of testosterone in the short-term.

**Supplementary Information:**

The online version contains supplementary material available at 10.1007/s00223-024-01296-z.

## Introduction

Transgender and gender diverse (TGD) people experience a gender identity which is different from the sex assigned at birth [1]. TGD assigned female at birth (*t*-AFAB) individuals can ask for gender-affirming hormone therapy (GAHT) [2] in order to obtain a certain degree of virilization and/or defeminization. Among these people, GAHT is mainly represented by testosterone (*T*) formulations. T therapy can be administered via transdermal, short or long-term intramuscular injections, depending on the person’s preferences and their clinical characteristics. The phenotypic effects of T are related to the wide distribution of the androgen receptor: they induce, over time, an increased growth of body and facial hair, greater muscle mass, male body contour, secondary amenorrhea, increased sexual desire, and clitoral growth [3]. On the other hand, the systemic effects of GAHT on organ metabolism and function are still to be fully elucidated both from a short- and long-term point of view.

In this context, bone health represents a pivotal topic to be discussed. Numerous studies have demonstrated how sex hormones impact bone metabolism in cisgender population [4–7]. Estrogens promote bone accrual during growth and maintain bone mass in adulthood through an upregulation of osteoblasts proliferation and the apoptosis of osteoclasts via RANKL and osteoprotegerin [8]. Conversely, cisgender men exhibit a hormonal profile characterized by low serum estradiol concentrations and high *T* levels [9]. Thanks to the ubiquitous expression of the aromatase enzyme, comprehending the skeletal tissue, a partial conversion of *T* into estrogens is attainable, thereby contributing to the maintenance of optimal bone health in this population [10].

A growing body of evidence has progressively analyzed the relationship between GAHT and bone health in *t*-AFAB individuals, who will be addressed as transgender men (TM) from now on [11]. This is a crucial aspect, in particular regarding TGD adolescents and young adults who start GAHT prior to attaining the peak bone mass [12], with potential consequences on fracture risk in later stages of life [13]. The available literature has mainly focused on bone mineral density (BMD) variations induced by GAHT. TM before starting GAHT seem to present a normal BMD, with osteoporosis rates similar to the general population. After starting GAHT, TM seemed to show a substantial stability of BMD up to 2 years of testosterone; this finding was also summarized by a previous meta-analysis by Figuera et al. [14]. Nevertheless, the objective of our study was to delve deeper, adding the latest literature and examining bone health assessment in TM from a more comprehensive perspective. Indeed, our intention was to incorporate serum parameters such as calcium, phosphate, parathyroid hormone (PTH), 25-hydroxyvitamin D (25OHD) and bone turnover markers (BTM).

Therefore, the aim of this systematic review and meta-analysis was to synthesize the current evidence on the short-term (up to 24 months) effects of T-based GAHT on BMD, serum parameters and BTM.

## Materials and Methods

The study was conducted according to the Preferred Reporting Items for Systematic Review and Meta-Analysis Protocols (PRISMA-P) [15]. It also complies with the guidelines of Meta-Analyses and Systematic Reviews of Observational Studies (MOOSE) [16]. The study is registered in the PROSPERO (International Prospective Register of Systematic Reviews) database, with the identification number CRD42024540037.

### Systematic Search Strategy

A systematic search was conducted in PubMed, Scopus, Web of Science, and Cochrane Library in order to identify all relevant English-language studies published on this topic through April 2024. For the extraction of publications the subsequent terms were used: “transgender”, “FtM”, “female to male”, “trans men”, “transgender men”, “transmen”, “AFAB”, “t-AFAB”, “testosterone”, “gender affirming hormone therapy”, “GAHT”, “androgen”, “bone*”, “bone mass density”, “BMD”, “DEXA”, “hip”, “lumbar”, “femoral neck”, “bone metabolism markers”, “bone turnover markers”, “calcium”, “phosphate”, “vitamin D”, “25OHD”, “PTH”, “parathormone”, “Procollagen type 1 N-terminal propeptide”, “P1NP”, “bone alkaline phosphatase”, “osteocalcin”, “1CTP”, “carboxy-terminal telopeptide of type 1 collagen”, “c-terminal telopeptide of type 1 collagen”, “CTx”. To combine these key terms, Boolean AND/OR operators were used. Finally, eligible studies were identified through a systematic search, supplemented by a manual search of references cited in the retrieved articles. Full texts were obtained for studies with unclear relevance based on the abstracts. The obtained reference lists were also scrutinized to find possible additional pertinent studies.

### Inclusion Criteria

The article selection process was carried out in several stages. In the first identification phase, database querying determined potentially eligible studies to include in the meta-analysis. Following the removal of duplicated articles tracked across multiple databases, in the second phase potential eligible papers were screened by reading their title and abstract. In the third phase, the remaining articles were evaluated in full-text for eligibility. Both prospective and retrospective observational studies, as well as longitudinal intervention studies, were deemed eligible, while non-experimental descriptive studies, studies conducted in populations other than the one of interest, studies in which endpoints other than those under analysis were evaluated, those with experimental designs other than the one of interest, and studies with incomplete or inaccurate data were excluded. The full-text of all selected studies was evaluated to determine their eligibility. The PRISMA flow-chart [17] was used to schematize the steps of article inclusion.

### Quality Assessment

The methodological quality of the included studies was assessed using the Effective Public Health Practice Project (EPHPP) Quality assessment tool [18]. This assessment tool, used for intervention studies such as randomized controlled trials and case–control studies, has been validated for use in systematic reviews as well [19]. The tool considers the following domains: selection bias, study design, confounding factors, study blindness, data collection method, and loss to follow-up. The quality of each domain can be reported as strong (strong), moderate (moderate) or weak (weak), and in the overall judgment, the quality can be reputed as “strong” if no weak score was assigned, “moderate” if only a weak judgment was assigned to one of the domains, and finally “weak” if two or more weak judgments were assigned to multiple domains.

### Data Extraction

To minimize bias and ensure the reliability of the review process, two independent reviewers (D.T. and L.M.) were involved in study selection, data extraction, and quality assessment. Any discrepancies were resolved through discussion or consultation with a third reviewer (J.V.).

The primary outcome was to evaluate the differences in lumbar spine, hip, femoral neck and whole-body BMD values before and after one and two years of GAHT with various types of T formulations among TM.

The secondary outcomes were to evaluate the differences of bone metabolism-related parameters, before and after one and two years of GAHT among TM. The investigated biomarkers were the following: calcium, phosphate, PTH, 25OHD, bone-specific alkaline phosphatase (BPA), osteocalcin (OC), procollagen 1 intact n-terminal pro-peptide (P1NP) and C-telopeptide of type 1 collagen (CTx).

Additional information extracted was first author, year of publication, country/geographical region, study design, sample size, mean age, BMI and ethnicity, type of T therapy used, duration of follow-up in months, and parameters investigated in the study.

### Statistical Analysis

Changes in BMD values were assessed by calculating mean differences (MD), while standardized mean difference (SMD) was used for metabolic and turnover markers. In the presence of significant heterogeneity, data were combined using random effects models, which assumed that the included studies have varying effect sizes, thus providing a conservative estimate of the overall effect [20]. For nonsignificant heterogeneity, the results were pooled in a fixed effects model.

Publication biases were evaluated by the funnel plot graph [21]. The funnel plot was also subjected to Duval and Tweedie trim-and-fill test, to help detect presumed missing studies to rebalance the funnel distribution in the presence of a skewed shape. In addition, this analysis recalculates the combined estimate considering these putative identified studies corrected for publication bias [22].

To investigate potential moderators (covariates) and to examine the associations between the covariates and the outcomes, meta-regression analyses were conducted. Age and BMI before GAHT, and serum testosterone concentrations before and after 12 and 24 months of GAHT were investigated as potential moderators. A *p*-value < 0.05 is considered statistically significant.

For both primary and secondary outcomes, Cochran's Chi-square test (Cochran's Q) and *I*^2^ test were used for the purpose of analyzing statistical heterogeneity between the outcomes of different studies considering a value of *I*^2^ ≥ 50% and/or a value of *P* < 0.05 indicative of significant heterogeneity [23].

Data analysis was performed using the *R* statistical software equipped with the *metafor* package (version 3.6.3, 2020; The *R* Foundation for Statistical Computing, Vienna, Austria).

## Results

### Selection of Studies

**Fig. 1:**
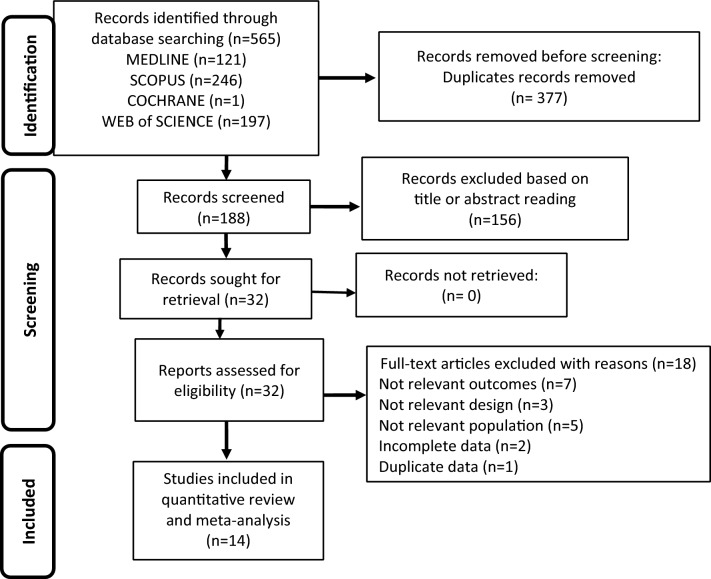
Flow chart of the study selection process

The search strategy queried 565 studies. Removal of duplicates resulted in 188 remaining studies, of which 156 were found to be irrelevant by screening the title and the abstract. Thus, 32 articles were identified; of them, 14 met the inclusion criteria (Chavaengkiat et al. [24]; Fernandez & Tannock [25]; Gava et al. [26]; Haraldsen et al. [27]; Meriggiola et al. [28]; Mueller et al. [29]; Pelusi et al. [30]; Turner et al. [31]; van Caenegem et al. [32]; van Kesteren et al. [33]; van Kesteren et al. [34]; Vlot et al. [35]; Wiepjes et al. [36]; Wiepjes et al. [37]). Figure 1 represents the flow-chart of the study selection process, while Table 1 summarizes the main characteristics of the included studies.

**Table 1:** Main characteristics of the included studies

Author	Year	Country	Study design	*N*	Mean age (years)	Mean BMI	Testosterone formulation	Follow-up (months)	DEXA parameters	Metabolism markers	Turnover markers
Chavaengkiat	2023	Thailand	Prospective	20	27 ± 7	22 ± 6	TE (im)	6	–		P1NP OC CTx
Fernandez & Tannock	2016	USA	Retrospective	19	27 (15–42)	28 ± 2	not specified (im)	3–6	–	Calcium	–
Gava	2021	Italy	Prospective	16	35 ± 6	23 ± 3.5	TU (im)	13.5	LS-BMD; HIP-BMD; TOTAL-BMD		BAP OC
Haraldsen	2007	Norway	Prospective	21	25 ± 5	NA	TE (im)	12	–	–	BAP OC
Meriggiola	2008	Italy	Prospective	15	34 ± 4	22 ± 2	TU (im)	13.5	LS-BMD	Calcium phosphate PTH	BAP OC
Mueller	2010	Germany	Prospective	45	30 ± 9	24 ± 4.5	TU (im)	24	LS-BMD; FN-BMD	–	–
Pelusi (a)	2014	Italy	Prospective	15	31 ± 3	22 ± 2	TE (im)	13.5	LS-BMD; TOTAL-BMD	Vitamin D PTH	BAP OC
Pelusi (b)	2014	Italy	Prospective	15	29 ± 3	24 ± 3	TG	13.5	LS-BMD; TOTAL-BMD	Vitamin D PTH	BAP OC
Pelusi (c)	2014	Italy	Prospective	15	28 ± 3	22 ± 2.5	TU (im)	13.5	LS-BMD; TOTAL-BMD	Vitamin D PTH	BAP OC
Turner	2004	USA	Prospective	8	37 ± 3	NA	TC (im) or TE (im)	24	LS-BMD; FN-BMD	–	
van Caenegem	2015	Belgium	Prospective	23	27 ± 9	24.5 ± 5	TU (im)	12	LS-BMD; HIP-BMD; FN-BMD; TOTAL-BMD	Vitamin D	CTx
van Kesteren	1996	Netherlands	Prospective	35	25 (15–35)	24 ± 12	TE (im), TU (oral)	12	LS-BMD	–	OC
van Kesteren	1998	Netherlands	Prospective	19	25 (15–34)	22 ± 7	TE (im), TU (oral)	28–63	LS-BMD	Calcium phosphate	OC
Vlot	2019	European multicentric	Prospective	132	24 (16–28)	25 ± 1.5	TG, TE (im), TU (im)	12	LS-BMD; HIP-BMD; FN-BMD	Vitamin D	P1NP CTx
Wiepjes	2017	Netherlands	Retrospective	543	25 (16–29)	26 ± 6	TG, ET, TU (im)	12	LS-BMD; HIP-BMD; FN-BMD	–	–
Wiepjes	2019	Netherlands	Retrospective	543	25 (16–29)	26 ± 6	TG, ET, TU (im)	24	LS-BMD	–	–

Age and BMI are expressed as media ± DS or median (25–75 interquartile range)

*BAP* bone alkaline phosphatase, *BMI* body mass index, *CTx* carboxy-terminal telopeptide of type 1 collagen, *ET* testosterone esters, *FN* femoral neck, *LS* lumbar spine, *NA* not available, *OC* osteocalcin, *P1NP* procollagen type 1 amino-terminal propeptide, *TC* testosterone cypionate, *TE* testosterone enanthate, *TG* testosterone gel, *TU* testosterone undecanoate

### Assessment of Study Quality

The quality assessment based on the EPHPP is represented in Table 1, Supplementary Material. Overall, most studies (12 out of 14) received a methodological quality rating of ‘‘moderate’’ [24, 26–36] and two studies were labeled as ‘‘weak’’ [25, 36]. The items ‘‘confounders’’ and “data collection methods” received the highest rating among all the included studies; on the contrary, the item ‘‘blinding’’ was the most lacking, as in none of the studies the participants and the research staff who assessed outcomes were blind to the study conditions. Two studies received a ‘‘weak’’ methodological quality rating regarding ‘‘withdrawals and dropouts’’, due to the large difference in the number of participants between initial enrollment and the end of follow-up [25, 37].

### Summary of Results

The overall studied subjects were 1484 TM. The represented population was mostly under 40 years of age and mean body mass index (BMI) ranged from 22 to 28 kg/m^2^.

Regarding the formulation of GAHT, enrolled individuals were treated with intramuscular (i.m.) mixed T esters in 2 studies [36, 37], i.m. T enanthate in 7 studies [24, 27, 30, 31, 33, 34], i.m. T cypionate in 1 study [31], T gel in 4 studies [30, 35–37], i.m. T undecanoate in 8 studies [26, 28–31, 35–37] while oral T undecanoate in 2 studies [33, 34] (Table 1).

### Primary Outcomes

**Fig. 2:**
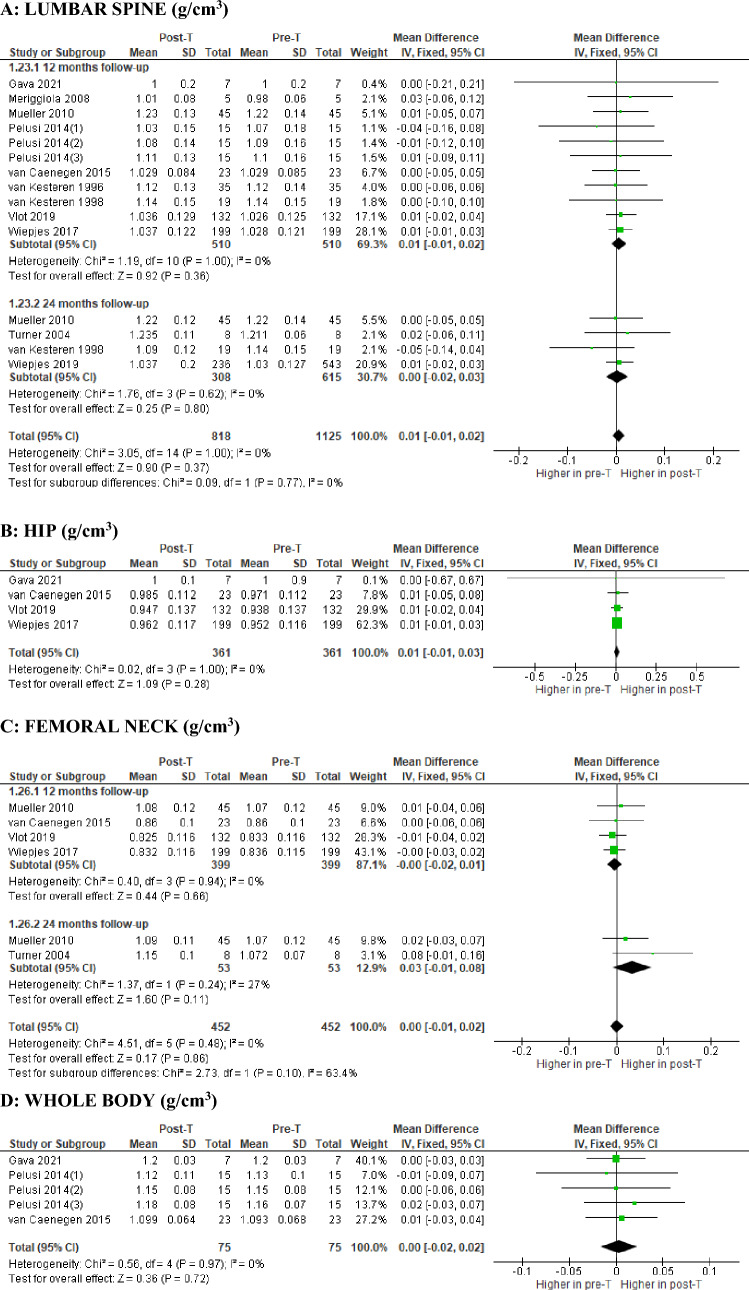
Forest plot of the effects of T-based GAHT on BMD in TM***.*** Diamonds indicate the overall effect estimates (and diamond width the 95% CI); squares indicate the weight of individual studies in the aggregate estimate. *CI* confidence interval, *IV* inverse variance, *T* Testosterone, *GAHT* gender-affirming hormone therapy, *TM* transmen

As shown in Fig. 2A, nine studies (eight prospective and one retrospective) analyzed the change in lumbar spine bone mass values after one year of GAHT and three studies (all prospective) after two years of GAHT: the difference between the aggregate means (MD) showed no significant change, in the absence of heterogeneity, in both the former on 510 subjects (MD 0.01 g/cm^3^; 95% CI: − 0.01, 0.02; *p* = 0.36; *I*^2^ = 0%, *P*_forheterogeneity_ = 1.00) and the latter on 308 subjects (MD 0.00 g/cm^3^; 95% CI: − 0.02, 0.03; *p* = 0.80; *I*^2^ = 0%, *P*_forheterogeneity_ = 0.62).

The absence of a significant difference on BMD between pre and post GAHT evaluations, in the absence of heterogeneity, was also confirmed at the hip after one year of GAHT in 361 subjects (MD 0.01 g/cm^3^; 95% CI: − 0.01, 0.03; *p* = 0.28; *I*^2^ = 0%, *P*forheterogeneity = 1.00)(Fig. 2B), at femoral neck after one year on 399 subjects (MD − 0.00 g/cm^3^; 95% CI: − 0.02, 0.01; *p* = 0.66; *I*^2^ = 0%, *P*forheterogeneity = 0.94) and after two years in 53 subjects (MD 0.03 g/cm^3^; 95% CI: − 0.01, 0.08; *p* = 0.11; *I*^2^ = 27%, *P*forheterogeneity = 0.24) (Fig. 2C), and at the whole-body after one year in 75 subjects (MD 0.00 g/cm^3^; 95% CI: − 0.02, 0.02; *p* = 0.72; *I*^2^ = 0%, *P*forheterogeneity = 0.97) (Fig. 2D).

**Fig. 3:**
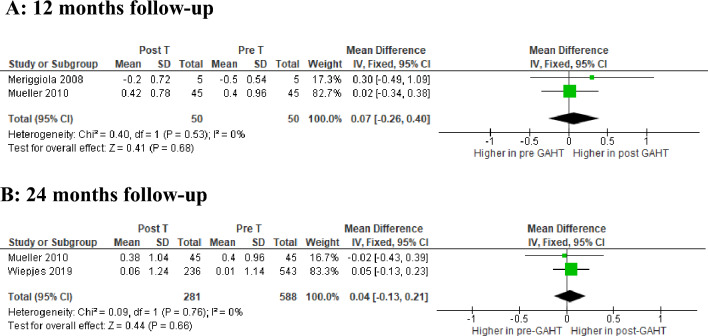
Forest plot of the effects of T-based GAHT on lumbar spine BMD z-score in TM***.*** Diamonds indicate the overall effect estimates (and diamond width the 95% CI); squares indicate the weight of individual studies in the aggregate estimate. *CI* confidence interval, *IV* inverse variance***,*** *T* testosterone, *GAHT* gender-affirming hormone therapy, *BMD* bone mineral density, *TM* transmen

Finally, there are no differences between the values highlighted in the TM and the standard references at the same age, biological sex and ethnicity at both 12 and 24 months as shown in Fig. 3A and Fig. 3B, respectively.

### Secondary Outcomes

As shown in Supplementary materials, no significant differences were found among the analyzed bone metabolism-related parameters. Calcium (Supplementary Fig. 1A), phosphate (Supplementary Fig. 1B), 25OHD (Supplementary Fig. 1C) and PTH (Supplementary Fig. 1D), did not appear to change significantly at the end of the follow-up.

**Fig. 4:**
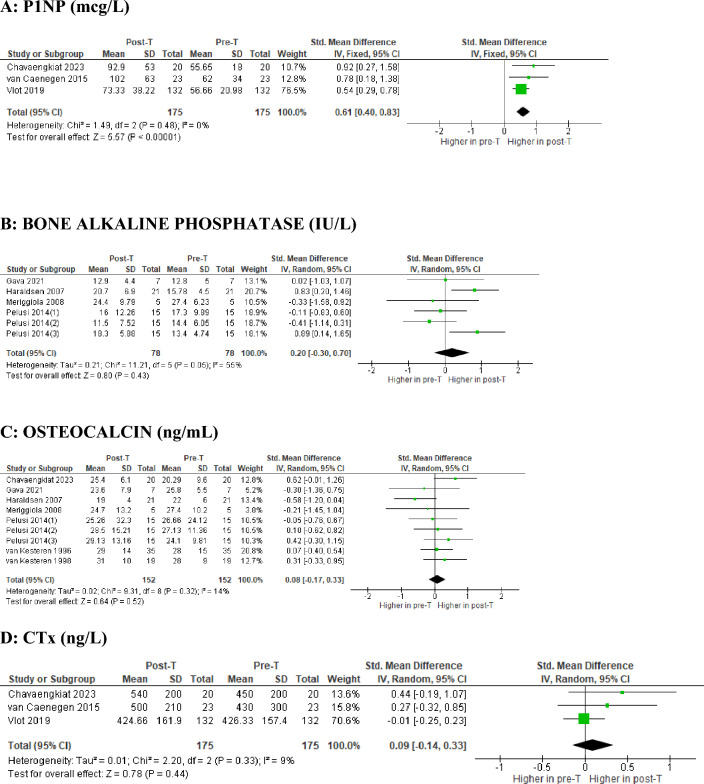
Forest plot of the effects of T-based GAHT on BTM in TM. Diamonds indicate the overall effect estimates (and diamond width the 95% CI); squares indicate the weight of individual studies in the aggregate estimate. *CI* confidence interval, *IV* inverse variance, *T* testosterone, *GAHT* gender-affirming hormone therapy, *BMD* bone mineral density, *TM* transmen

Regarding BTM, as shown in Fig. 4A, three prospective studies, including a total of 175 TM, highlighted noteworthy changes in P1NP after 12 months of T therapy: the difference between MD showed a statistically significant increase, in absence of heterogeneity (SMD 0.61 mcg/l; 95% CI: 0.40–0.83; *p* < 0.0001; *I*^2^ = 0%, *P*forheterogeneity = 0.48). On the contrary, none of the other BTM analyzed appeared to change significantly at the end of the follow-up, including BAP (Fig. 4B), OC (Fig. 4C) and CTx (Fig. 4D).

## Sensitivity Analysis

### Publication Bias

**Fig. 5:**
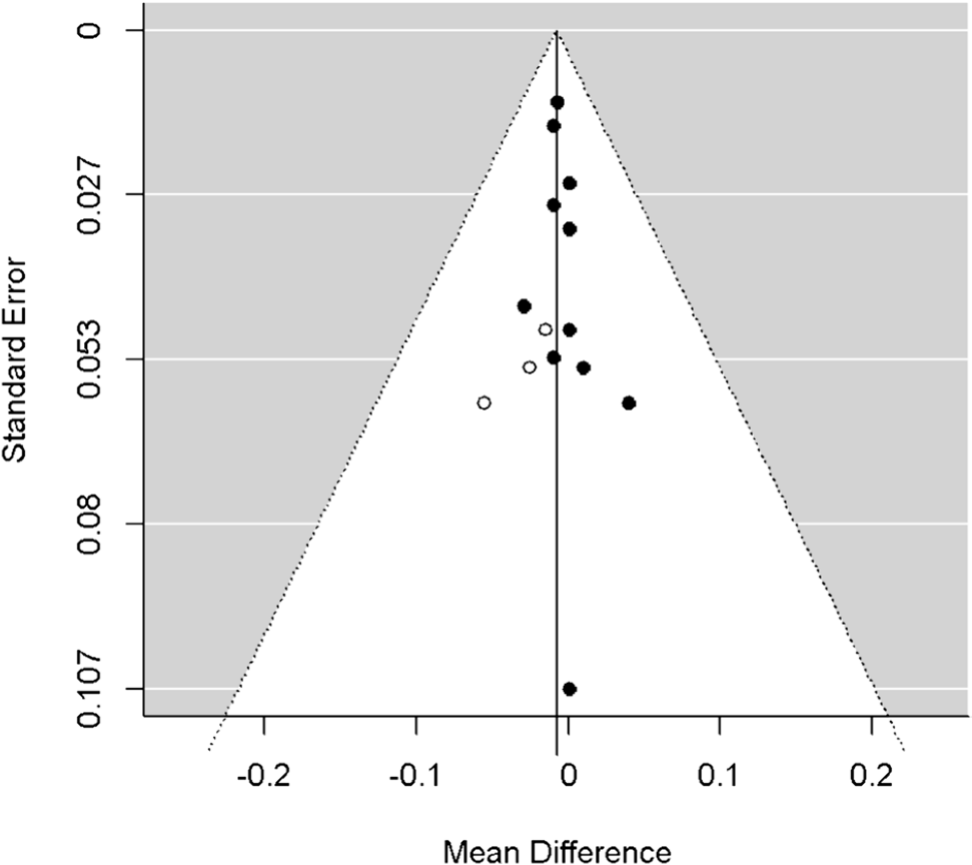
Funnel plot and trim-and-fill test of lumbar spine BMD studies

As shown in Fig. 5, the rather asymmetrical funnel plot shapes of the analyses of lumbar spine BMD studies could have suggested the presence of publication bias. Indeed, the trim-and-fill analysis identified three putative missing studies on the left side of the distribution, but correction for publication bias by including these studies in the analysis had little effect on the overall estimate (adjusted MD: − 0.01; 95%CI: − 0.02, 0.01; *p* = 0.3).

### Meta-regression Analyses

Meta-regression analyses revealed no significant correlations between final lumbar spine BMD and the age at GAHT initiation (*β* = 15.37; 95% CI: − 18.70 to 49.43; *p* = 0.3766), baseline serum T (*β* = 3.08; 95% CI: − 7.96 to 14.12; *p* = 0.5844) and BMI (*β* =  − 1.47; 95% CI: − 3.15 to − 0.21; *p* = 0.0859) or pre/post GAHT changes in BMI (*β* =  − 2.44; 95% CI: − 11.55 to 6.66; *p* = 0.5990) or serum T levels (*β* = 0.23; 95% CI: − 1.38 to 1.85; *p* = 0.7776).

## Discussion

The present study aimed to meta-analyze the effects on BMD, serum bone parameters and BTM among TM starting T-based GAHT. GAHT aims to improve the quality of life of TGD people, aligning the experienced gender with the assigned sex at birth [2]. In this context, the available data regarding short- and long-term effects of GAHT on bone health are not always conclusive.

First, regarding BMD evaluations, this study showed that after one and two years of T-based GAHT, no significant changes were reported at lumbar spine, hip, femoral and whole-body assessments among TM, even in relation to standard references for the same age, sex at birth, and ethnicity.

Accumulating evidence from basic and translational studies underscores the pivotal role estrogens play in supporting bone microarchitecture and skeletal scaffolding across the lifespan. Alpha and beta estrogen receptors and androgen receptors are represented in bone and bone marrow [10]. The post-menopausal lowering of estrogen levels brings bone loss in the trabecular and cortical bone [38]. Even in cisgender men, a decrease in serum T values is directly associated with bone loss. This event is even worsened by the lowering of aromatase-derived estradiol [9, 39]. Other models have been used to assess how impactful sex hormones are on bone health, such as cisgender men with aromatase gene deficiency or cisgender women taking aromatase inhibitors for breast cancer; the evidence derived from these subgroups further support the pivotal role that circulating estrogens play in promoting bone health [40, 41]*.* It has to be considered that part of bone metabolism is related to muscle-induced mechanical load on the skeleton. Muscle mass lowers and fat mass increases in hypogonadal cisgender men and women and this may be an adjunctive detrimental factor in the maintenance of proper bone health [42, 43].

In this context, TM represents a unique model to study bone metabolism. In fact, due to T-based GAHT, estrogen levels decrease to the cisgender male reference, while body mass composition significantly differs from cisgender women [3]. DXA-derived evaluations of lumbar, hip, femoral and whole-body BMD did not show significant change after 12 and 24 months of T-based GAHT. A recent meta-analysis on anthropometric and metabolic changes in t-AFAB people on T showed that while estrogen levels lower, an increase in lean mass is reported, up to 4.12 kg for TM after one year of GAHT [44]. This may induce a new balanced environment, supportive in maintaining a proper BMD. The data reported by our work were consistent with what has already been reported in literature. In fact, most studies, including a meta-analysis which analyzed prospective and cross-sectional studies conducted in TGD people on GAHT, did not report changes in areal BMD of the spine, total hip, or femoral neck during GAHT in the short-term when also compared to cisgender population [27, 29–34].

Second, serum parameters related to bone metabolism, such as calcium, phosphate, PTH, and 25OHD did not differ significantly after starting GAHT. Bone is mostly composed by hydroxyapatite, a mineral who contains calcium and phosphate [45]; its tissue is constantly remodeled, releasing these two ions under the control of 25OHD and PTH [46]. Due to important changes in the hormonal milieu induced by GAHT, a variation of these serum parameters might be speculated. Nevertheless, the effects of sex steroids on calcium and phosphate metabolism are not totally clear. In fact, studies conducted on cisgender men and on male animal models highlighted a higher urinary excretion of calcium, while studies regarding hypogonadal cisgender men on hormone replacement therapy or androgen-deprived men showed inconsistent results [45]. Estrogens deficiency as in postmenopausal cisgender women is associated with hypercalcemia and hypercalciuria and estrogen replacement therapy restores the previous state [47, 48]. On the other hand, phosphate levels seem to be indirectly related with T serum levels [49], and T deprived men showed higher phosphate levels and phosphate renal reabsorption, along with an increase in PTH levels [50]. Estrogen levels in both cisgender men and women showed an indirect correlation with phosphate levels and lower phosphate reabsorption [51, 52]. In reference to 25OHD concentrations, they are mostly positively related to *T* levels [53–56] in cisgender men, while in cisgender women a consistent correlation has hardly been found [57, 58]. Given the aforementioned points, variations among these parameters could have been expected in TM starting GAHT. However, in our meta-analysis, no changes in these factors were identified, although a trend towards higher levels of 25OHD seems to be emerging, consistent with what has been shown in another study [59]. One potential explanation for this phenomenon may be linked to the utilization of vitamin D supplements. In two out of three articles included in this meta-analysis [30, 35] no vitamin D supplementation was taken immediately before or during the studies. In the other one [32], vitamin D supplements were prescribed to nine TM (40%) who had basal 25OHD serum levels < 20 ng/ml but, after one year, only two subjects were still using vitamin D supplements. Thus, the potential impact on 25OHD average values is likely to be negligible. In addition, as described by Chen et al. [59], in a population of 30 TM on GAHT, the circulating concentration of vitamin D binding protein tended to slightly decrease after three months, while the concentrations of free and bioavailable 25OHD tended to be higher, however in a non-statistically significant fashion.

Lastly, BTM remained stable among TM during the first two years after starting GAHT, except for P1NP. Studies among cisgender hypogonadal men on hormonal replacement therapy highlighted a decline in bone resorption markers, while bone formation parameters increased [60, 61]. This study produced similar results deriving from the analysis of three studies including 175 TM. In fact, P1NP showed a significant increase over time. P1NP is a collagen derived marker and represents a hallmark for bone formation; this parameter is of particular sensitivity and is therefore especially useful for monitoring bone anabolic therapies. *T*-based GAHT aims to establish a new hormonal milieu, embodied by the reduction of endogenous estradiol induced by ovarian function suppression, consequent to increasing levels of exogenous *T*. The increase of P1NP after the first months of *T*-based GAHT seems to indirectly show a positive effect of *T* on bone metabolism of TM. Androgens induce a change in bone geometry by promoting a subperiosteal expansion and cortical thickness, [62]. Eventually, maintaining serum *T* levels in a range similar to cisgender men may mitigate the negative effect of estrogen reduction on bone. Compliance with and adequate delivery of GAHT represent paramount factors to properly establish and maintain an optimal hormonal environment, and consequently supporting an adequate BMD [63]. This aspect should be emphasized during consultations to further support bone health in TGD population.

### Strengths and Limitations

The valuable aspects of our study are the great sample size, the largest among all reviews to date, and the rigorous selection of studies included in the quantitative analysis, characterized by the absence of dropouts, at least regarding 12 months available data: methodological accuracy in the inclusion criteria may underline the negligible heterogeneity that emerged in our analyses.

This meta-analysis presents some limitations. First, the observational design of all the included studies did not include a control group; therefore, it is impossible to determine whether some of the observed effects are related to T therapy or to other factors. Furthermore, a pivotal piece of information would have been determining the impact of different T formulations on BMD values across different body sites. Unfortunately, as shown in Table 1, only a small number of studies that evaluated the same endpoint used the same type of T in unmixed protocols. This made it impossible to conduct subgroup analyses based on the different formulations. Lastly, lack of proper data precluded the assessment of variations in fracture risk within this population.

## Conclusions

This systematic review and meta-analysis intended to evaluate BMD, serum bone parameters and BTM after the initial 12 and 24 months of T-based GAHT in TM. The use of T in TM appears to induce bone formation, as indicated by P1NP levels. However, no significant changes were observed in BMD, calcium, phosphate, 25OHD, PTH, or other analyzed BTM. Meta-analytic studies on longer term effects of T-based GAHT on bone health will be necessary to properly address its safety among TM.

## Supplementary Information

Below is the link to the electronic supplementary material.

Supplementary file1 (DOCX 109 KB)
