# Supplementary material for: Short-Term Changes in Bone Metabolism Among Transgender Men Starting Gender-Affirming Hormone Therapy: A Systematic Review and Meta-analysis
Source: Calcif Tissue Int. 2024 Oct 2;115(5):624–35. doi: 10.1007/s00223-024-01296-z (PMC11531450; doi:10.1007/s00223-024-01296-z)
Supplement: Supplementary file 1 — Supplementary file1 (DOCX 109 KB) [file 223_2024_1296_MOESM1_ESM.docx]

**SUPPLEMENTARY MATERIAL**

**Supplementary Figure 1.** Forest plot of the effects of T-based therapy on bone metabolism markers

**A: CALCIUM (mg/dL)**

**
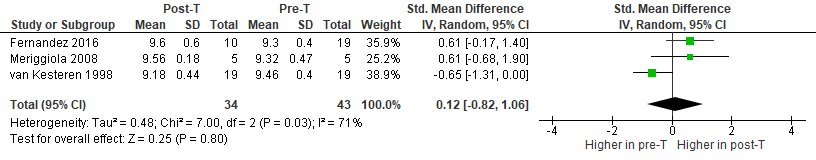
**

**B: PHOSPHATE (mmol/L)**

**
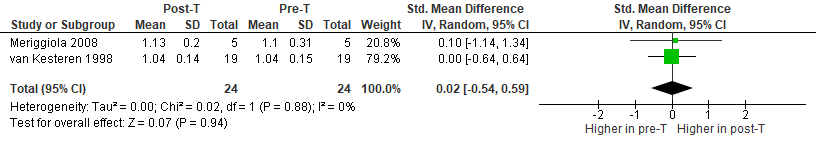
**

**C: 25-HYDROXYVITAMIN D (ng/mL)**

**
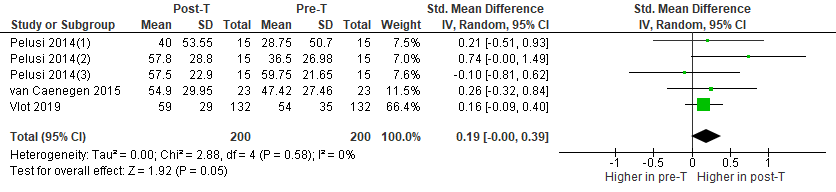
**

**D: PARATHORMONE (pg/mL)**

**
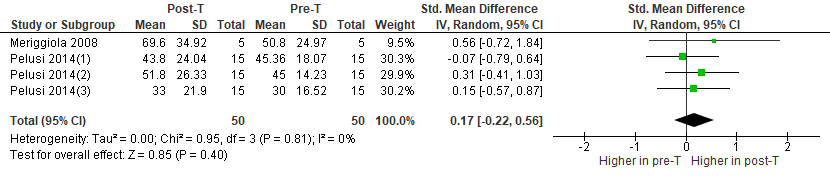
**

Abbreviations: T: testosterone, GAHT: Gender Affirming Hormone Therapy, TM: transmen

**Supplementary Table 1.** Quality assessment of included studies by EPHPP

| **Study** | **Selection Bias** | **Study Design** | **Confounders** | **Blinding** | **Data Collection Methods** | **Withdrawals and Drop-Outs** | **Global Rating** |
| --- | --- | --- | --- | --- | --- | --- | --- |
| *Chavaengkiat 2023* | moderate | moderate | strong | weak | strong | strong | moderate |
| *Fernandez & Tannock 2016* | moderate | moderate | strong | weak | strong | weak | weak |
| *Gava 2021* | moderate | moderate | strong | weak | strong | strong | moderate |
| *Haraldsen 2007* | moderate | moderate | strong | weak | strong | strong | moderate |
| *Meriggiola 2008* | moderate | moderate | strong | weak | strong | strong | moderate |
| *Mueller 2010* | moderate | moderate | strong | weak | strong | strong | moderate |
| *Pelusi 2014* | moderate | moderate | strong | weak | strong | strong | moderate |
| *Turner2004* | moderate | moderate | strong | weak | strong | strong | moderate |
| *van Caenegem 2015* | moderate | moderate | strong | weak | strong | strong | moderate |
| *van Kesteren 1996* | moderate | moderate | strong | weak | strong | strong | moderate |
| *van Kesteren 1998* | moderate | moderate | strong | weak | strong | strong | moderate |
| *Vlot 2019* | moderate | moderate | strong | weak | strong | strong | moderate |
| *Wiepjes 2017* | moderate | moderate | strong | weak | strong | strong | moderate |
| *Wiepjes 2019* | moderate | moderate | strong | weak | moderate | weak | weak |
